# Targeting the Gut–Brain Axis: Pharmacological Modulation of the Microbiome for Neurological and Behavioural Disorders in Companion Animals

**DOI:** 10.1002/vms3.71105

**Published:** 2026-07-22

**Authors:** Sakineh Khanamani Falahatipour, Motahareh Soltani

**Affiliations:** ^1^ Department of Comparative Biosciences Faculty of Veterinary Medicine University of Tehran Tehran Iran; ^2^ Pistachio Safety Research Center Rafsanjan University of Medical Sciences Rafsanjan Iran

**Keywords:** companion animals, gut–brain axis, microbiome, neurological disorders, psychobiotics

## Abstract

**Background:**

The gut–brain axis (GBA) represents a paradigm shift in veterinary neuropharmacology, offering novel approaches for managing neurological and behavioural disorders in companion animals.

**Objectives:**

This review synthesizes current evidence on the bidirectional communication between the gut microbiome and the central nervous system, examining the neural, endocrine, immune, and metabolic pathways that facilitate this dialogue. We explore the unique aspects of canine and feline microbiomes and their implications for species‐specific drug development and critically evaluate emerging pharmacological strategies, including psychobiotics, prebiotics, synbiotics and faecal microbiota transplantation (FMT), highlighting their clinical applications in conditions ranging from anxiety and aggression to cognitive dysfunction and epilepsy.

**Methods:**

This narrative review followed established guidelines for evidence synthesis in veterinary medicine. A comprehensive literature search was performed using PubMed, Google Scholar and Scopus databases covering publications from January 2011 to March 2026.

**Results:**

While promising results have been demonstrated with specific strains, such as *Bifidobacterium longum* BL999 and *Lactiplantibacillus plantarum* PS128, significant challenges remain. These include methodological limitations in microbiome research, the predominance of correlative over causal evidence and the need for standardized diagnostic tools.

**Conclusions:**

Future directions must prioritize large‐scale longitudinal studies, robust clinical trials and advanced multi‐omics approaches to establish causal mechanisms and develop personalized, microbiome‐targeted therapies. Realizing this potential requires a shift from correlative data to causal mechanisms, from a one‐size‐fits‐all approach to species‐specific therapeutics and from rodent models to rigorous trials in dogs and cats.

## Graphical abstract

Gut microbiota communicate with the brain via the vagus nerve, SCFAs, GABA and serotonin, while maintaining gut barrier integrity and reducing neuroinflammation. In dogs, psychobiotics and prebiotics show moderate evidence, FMT shows very low evidence (case reports only), and dietary evidence is growing, but feline data remain limited. These adjunctive therapies require large‐scale species‐specific trials before clinical adoption.

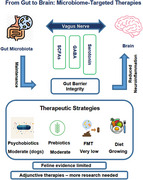

## 1 Introduction: The Gut–Brain Axis (GBA), a Paradigm Shift in Veterinary Neuropharmacology

The management of neurological and behavioural disorders in companion animals is undergoing a significant transformation, moving beyond conventional neurocentric approaches to embrace a more holistic understanding of pathophysiology. Central to this shift is the microbiota‐gut–brain axis (MGBA), a complex, bidirectional communication network that integrates the central nervous system (CNS), the enteric nervous system (ENS) and the vast community of gut microorganisms (Li et al. 2025). This continuous crosstalk occurs via neural pathways, such as the vagus nerve, endocrine signalling involving gut hormones and microbial metabolites and immune mechanisms, including cytokine modulation (Mayer et al. 2022). The gut microbiota is not a passive resident but an active mediator in this network, producing a repertoire of neuroactive compounds like short‐chain fatty acids (SCFAs) and neurotransmitters that fundamentally influence brain development, emotional regulation and cognitive function, thereby inextricably linking gastrointestinal health to neurological status.

### 1.1 Species‐Specific Microbiomes: Implications for Therapeutics

A critical consideration in veterinary medicine is the profound species‐specificity of the gut microbiome. Although dogs, cats and humans share dominant bacterial phyla, such as Firmicutes and Bacteroidetes, their compositional and functional profiles are distinct, shaped by evolutionary, dietary and physiological differences (Deng and Swanson 2015; Morelli et al. 2021). These differences are not merely taxonomic but have direct clinical and pharmacological consequences. For instance, species‐specific dysbiosis patterns are evident in diseases like chronic enteropathy in dogs, characterized by a decrease in key bacteria like *Faecalibacterium* and *Clostridium hiranonis*, which are crucial for bile acid metabolism (Suchodolski 2022). This impaired conversion disrupts gut homeostasis and alters the metabolic environment, which can directly impact drug metabolism and efficacy. Furthermore, microbiome‐derived metabolites like trimethylamine *N*‐oxide (TMAO) have been linked to species‐specific conditions, such as chronic heart failure in dogs and chronic kidney disease in cats (Suchodolski 2022). Consequently, a pharmacological agent's bioavailability, toxicity and overall therapeutic outcome can be unpredictably influenced by the host's unique microbial ecosystem, necessitating species‐tailored drug development and a move away from direct extrapolation from human models.

It is important to acknowledge that the vast majority of GBA research in companion animals has focused on dogs, with feline data remaining comparatively limited. As noted by McGrath et al. (2024), research into cognition and the impact of nutrition on cognitive health in cats ‘lags behind that in dogs’. Recent studies have documented the composition of the feline gut microbiota and its association with gastrointestinal and metabolic diseases (Shi et al. 2024; Zha et al. 2024). Emerging evidence has also identified gut dysbiosis in cats with chronic kidney disease (Summers and Quimby 2024) and metabolic disorders, including obesity and diabetes (Ji et al. 2023). Furthermore, bile acid dysmetabolism—specifically the loss of secondary bile acids due to depletion of *Peptacetobacter hiranonis*—has been implicated in feline neuroinflammation and cognitive dysfunction (Németh et al. 2026). However, direct evidence linking the feline gut microbiome to behavioural outcomes, such as anxiety, aggression and cognitive dysfunction, remains preliminary and largely extrapolated from canine and human studies. Throughout this review, we explicitly note when conclusions are drawn primarily from canine studies and caution against direct extrapolation to cats given their distinct obligate carnivore physiology.

### 1.2 The Rationale for Novel Therapeutic Modalities

The limitations of conventional psychotropic drugs, such as selective serotonin reuptake inhibitors (SSRIs) and benzodiazepines, underscore the urgent need for these novel approaches. Their use in veterinary practice is constrained by several factors: Significant side effects, including sedation and potential personality changes that concern owners; a lack of robust, species‐specific efficacy data; and often, a delay in onset of action during critical periods (van Haaften et al. 2020; Eagan et al. 2025; Sacoor et al. 2024). Perhaps most importantly, these conventional therapies primarily manage symptoms without addressing the underlying pathophysiology, which is increasingly implicated in disruptions of the GBA and associated microbial dysbiosis (Kiełbik and Witkowska‐Piłaszewicz 2024). This therapeutic gap has catalyzed the exploration of microbiome‐targeted interventions. Strategies, such as specific probiotic and prebiotic formulations, synbiotics and faecal microbiota transplantation (FMT), aim to restore microbial homeostasis and modulate the production of key neuroactive compounds and inflammatory pathways, offering a more fundamental and potentially safer alternative for managing behavioural and neurological disorders in dogs and cats (Sacoor et al. 2024; Kiełbik and Witkowska‐Piłaszewicz 2024).

### 1.3 Literature Search Strategy

This narrative review followed established guidelines for evidence synthesis in veterinary medicine (Sargeant and O'Connor 2020). A comprehensive literature search was performed using PubMed, Google Scholar and Scopus databases, covering publications from January 2011 to March 2026. The search strategy combined keywords related to (1) the GBA (‘GBA’, ‘MGBA’, ‘microbiome’), (2) companion animals (‘dog’, ‘canine’, ‘cat’, ‘feline’) and (3) behavioural or neurological outcomes (‘anxiety’, ‘aggression’, ‘cognition’, ‘epilepsy’, ‘behavioural disorder’, ‘cognitive dysfunction’). Boolean operators (‘AND’, ‘OR’) were used to combine search terms. Reference lists of retrieved articles were manually screened for additional relevant studies. Only English‐language peer‐reviewed articles were included. Given the narrative nature of this review, a formal risk of bias assessment of individual studies was not performed; however, the strength of evidence for each intervention was graded as described in Section 4.

### 1.4 Objective of This Review

This review aims to synthesize the current evidence on the MGBA in companion animals and critically evaluate the potential for its pharmacological modulation. We will propose a framework for developing and implementing these novel, microbiome‐targeted therapeutic strategies to improve the management of neurological and behavioural disorders in dogs and cats.

## 2 Mechanisms of Gut–Brain Communication in Companion Animals

The gut microbiome is a pivotal regulator of host physiology, and disruptions to its community structure can exert wide‐ranging effects on overall health. Maintaining homeostasis is dependent on a complex, interactive dialogue between the host and its microbiota, mediated by an array of multidirectional signalling molecules (El Aidy et al. 2016). This inter‐kingdom signalling extends beyond the gastrointestinal tract to influence distant organs, including the brain. Understanding the mechanistic principles of this communication is therefore fundamental for developing novel microbiome‐targeted strategies to improve mental health outcomes in companion animals.

The gut microbiome communicates with the brain through a sophisticated, integrated network of neural, endocrine, immune and metabolic pathways, which work in concert to maintain neurological and behavioural homeostasis in dogs and cats (Gershon and Margolis 2021; Kasarello et al. 2023; Gorzelanna and Miszczak 2024).

### 2.1 Neural Pathways: The Vagus Nerve

A primary route for direct neural communication is the vagus nerve. Its sensory afferents within the gut wall detect microbial metabolites and relay this information directly to the brainstem (Gershon and Margolis 2021). This nerve is not a simple conduit; it can differentiate between bacterial types, mediating both anxiogenic and anxiolytic effects (Forsythe et al. 2014). Furthermore, the vagus nerve plays an immunomodulatory role, participating in an ‘anti‐inflammatory reflex’ that can be triggered by gut signals to dampen systemic inflammation, thereby influencing brain function and mood (Forsythe et al. 2014).

### 2.2 Endocrine and Neuroendocrine Signalling

The endocrine system facilitates gut–brain crosstalk largely through specialized enteroendocrine cells (EECs), particularly enterochromaffin cells. These cells act as key transducers by releasing neurotransmitters and gut hormones in response to microbial cues (Gershon and Margolis 2021; Dong and Mayer 2024). A critical output is the synthesis and release of serotonin (5‐HT), a key neurotransmitter for mood regulation. Peptides, such as glucagon‐like peptide‐1 (GLP‐1) and peptide YY (PYY), are also released, influencing both local enteric reflexes and central processes (Kasarello et al. 2023). This signalling system is complemented by microbiome‐mediated modulation of the hypothalamic–pituitary–adrenal (HPA) axis, the body's central stress response system, thereby directly linking microbial balance to stress hormone release and emotional states (Gershon and Margolis 2021).

### 2.3 Immune and Inflammatory Mechanisms

Immune signalling represents a critical pathway through which the microbiome influences the brain. Immune cells within the gut express pattern recognition receptors, such as Toll‐like receptors (TLRs), which detect microbe‐associated molecular patterns (MAMPs). This interaction can initiate the release of cytokines, which may then trigger neuroinflammatory responses by impacting neurons and glial cells in the CNS (Gershon and Margolis 2021; Kasarello et al. 2023). Dysbiosis can exacerbate this process by compromising intestinal barrier integrity, leading to a ‘leaky gut’ that allows bacterial metabolites and inflammatory mediators to enter circulation, thereby provoking a systemic immune response that impacts brain function and behaviour (Homer et al. 2023).

### 2.4 Key Microbial Metabolites as Humoural Messengers

Microbial metabolites serve as indispensable humoural messengers in the GBA. SCFAs—such as butyrate, propionate and acetate—produced from dietary fibre fermentation are of paramount importance. SCFAs modulate host physiology by binding to free fatty acid receptors (FFARs) and by inhibiting histone deacetylases (HDACs), influencing gene expression, reducing neuroinflammation and supporting blood–brain barrier integrity (Kasarello et al. 2023; Grenham et al. 2011). Beyond SCFAs, gut bacteria directly synthesize neurotransmitters like GABA (e.g., by *Lactobacillus* and *Bifidobacterium* species) and metabolize dietary tryptophan into neuroactive derivatives, further modulating brain function and behaviour (Homer et al. 2023).

In summary, the continuous, multidirectional dialogue along the GBA, facilitated by these interconnected pathways, is essential for maintaining neurological and behavioural equilibrium in companion animals. Table 1 summarizes the key microbial metabolites involved in GBA signalling, their mechanisms of action and clinical implications. A breakdown in this complex communication, often stemming from gut dysbiosis, is a key factor in the pathogenesis of behavioural and neurological disorders.

**TABLE 1:** Key microbial metabolites and their roles in gut–brain axis signalling.

Metabolite	Producing bacteria	Mechanism of action	Neurological/Biological effects	Clinical implications
Short‐chain fatty acids (SCFAs)—butyrate, propionate, acetate	*Faecalibacterium*, *Clostridium*, *Bacteroides*	Bind to FFARs Inhibit HDACs Enhance gut barrier function	Reduce neuroinflammation Support BBB integrity Modulate neurotransmitter synthesis Regulate HPA axis	Cognitive function improvement Anxiety reduction Potential in epilepsy management
GABA	*Lactobacillus*, *Bifidobacterium*	Bind to GABA receptors Modulate neuronal excitability	Anxiolytic effects Sedative properties Anti‐convulsant effects	Anxiety disorders Stress‐related behaviours Seizure control
Serotonin (5‐HT) precursors	*Enterococcus*, *Escherichia*	Tryptophan metabolism 5‐HTP production	Mood regulation Appetite control Sleep‐wake cycle	Depression‐like behaviours Anxiety disorders Appetite regulation
Tryptophan derivatives	Multiple species	Activate AhR pathway Kynurenine pathway modulation	Immune regulation Neuroprotection/neurotoxicity balance	Inflammation modulation Neurodegenerative disorders

Abbreviations: FFARs, free fatty acid receptors; HDACs, histone deacetylases; HPA, hypothalamic–pituitary–adrenal.

## 3 Pharmacological Targets and Intervention Strategies

The GBA presents a novel therapeutic landscape for managing neurological and behavioural disorders in companion animals. By targeting the complex interplay between the gut microbiota and the CNS, a range of intervention strategies can be deployed to restore microbial homeostasis and mitigate neuropsychiatric symptoms.

### 3.1 Probiotics, Prebiotics and Synbiotics

Probiotics, defined as live microorganisms that confer a health benefit on the host, are a cornerstone of microbiome‐based therapeutics. In companion animals, specific bile‐resistant strains of *Lactobacillus* and *Bifidobacterium* are administered to enhance gastrointestinal integrity, reduce inflammation and protect against enteropathogens, thereby indirectly supporting neurological health (Shah et al. 2024). The efficacy of these interventions stems from their ability to modulate key host signalling pathways. As noted by Kaushik et al. (2024), prebiotics, probiotics and synbiotics can combat metabolic and inflammatory disorders by targeting critical mechanisms, including the AMPK and insulin signalling pathways for metabolic control and the NF‐κB pathway for inflammation reduction. By reshaping the gut microbiota and its metabolic output, these interventions represent a promising, personalized therapeutic strategy.

The timing of intervention is critical. Mikami et al. strongly advocate for a therapeutic strategy that administers probiotics and prebiotics during early developmental stages to prevent or mitigate pathological behaviours. Their research demonstrates that interventions are most effective early in development, as normalizing the gut microbiota at a young age can significantly reduce subsequent aggression, highlighting the importance of key pharmacological targets like neurotransmitter systems (dopamine and serotonin) and the HPA axis.

### 3.2 Microbial Metabolites as Key Targets

A primary mode of action for GBA‐targeted therapies is the modulation of microbial metabolites. Key pharmacological targets include SCFAs, tryptophan derivatives, bile acids and microbially synthesized neurotransmitters, which collectively modulate neuroinflammation, synaptic plasticity and blood–brain barrier integrity (Ortega et al. 2022). Probiotics function as an emerging therapeutic strategy by increasing the production of neuroprotective metabolites like butyrate to counteract pathological processes (Shokr et al. 2025). This approach advocates for a paradigm shift towards integrated, mechanism‐based treatments that holistically manage complex comorbidities.

### 3.3 Advanced and Adjunctive Interventions

Beyond probiotics and prebiotics, more advanced interventions are showing promise. FMT is an investigational approach with preliminary evidence in improving behavioural scores in conditions like autism spectrum disorder, suggesting its applicability for complex behavioural disorders in companion animals (Mikami et al. 2023). Furthermore, the therapeutic scope is expanding to include the gut mycobiome (the fungal community). Antifungal agents, specific probiotics like *Saccharomyces boulardii* and dietary modifications can restore mycobiome balance, strengthen intestinal integrity and reduce the neuroinflammation driven by fungal components, such as β‐glucans (Hadrich et al. 2025).

Dietary modifications serve as a powerful adjunctive strategy. Diets, such as the high‐fibre Mediterranean diet or the ketogenic diet, can profoundly reshape the microbial ecosystem and its metabolic output (Ortega et al. 2022; Hadrich et al. 2025). Adjunctive nutraceuticals, including omega‐3 fatty acids and polyphenols, further support microbial and metabolic balance, offering a holistic, multi‐pronged approach to managing GBA‐related disorders (Ortega et al. 2022). The future of this field lies in integrating multi‐omics and biomarker‐driven approaches to enable personalized, mechanism‐based treatments for companion animals. The current landscape of microbiome‐targeted interventions, their mechanisms and evidence levels are summarized in Table 2.

**TABLE 2:** Current and emerging microbiome‐targeted interventions in companion animals.

Intervention type	Specific examples	Mechanisms	Evidence level	Clinical applications
Psychobiotics	*Bifidobacterium longum* BL999, *Lactiplantibacillus plantarum* PS128	SCFA production Neurotransmitter regulation HPA axis modulation Cytokine reduction	Moderate evidence from small canine trials	Separation anxiety Aggression Fear responses
Prebiotics	FOS, GOS, XOS	Selective growth of beneficial bacteria Enhanced SCFA production	Moderate evidence	General anxiety Cognitive support Gut health maintenance
Faecal microbiota transplantation (FMT)	Donor‐screened faecal material	Microbial community restoration Metabolic pathway normalization Immune system modulation	Very low/Preliminary (case reports only)	Drug‐resistant epilepsy Severe behavioural disorders Chronic GI conditions
Specialized diets	Ketogenic diet, MCT‐enriched diet, high‐fibre diet	Microbial composition alteration Metabolite profile modification Inflammation reduction	Growing evidence	Cognitive dysfunction Epilepsy Age‐related behavioural changes
Synbiotics	*Lactobacillus* + FOS, *Bifidobacterium* + GOS	Combined probiotic‐prebiotic effects Enhanced microbial colonization	Emerging evidence	Comprehensive microbiome support Behavioural disorder prevention

*Note*: Evidence grades follow the framework of Sargeant et al. (2022) and Fletcher et al. (2024). ‘Moderate’ indicates some confidence in effect direction but further research likely to change the estimate. ‘Low’ indicates limited confidence. ‘Very low’ indicates the estimate is very uncertain, and clinical application is not yet recommended outside research settings.

Abbreviations: HPA, hypothalamic–pituitary–adrenal; MCT, medium‐chain triglyceride; SCFA, short‐chain fatty acid.

### 3.4 The Role of Diet and Animal Feed

Diet is the most modifiable factor shaping gut microbial composition in companion animals. Commercial pet foods vary in macronutrient profile, fibre type and functional ingredients, each of which can influence the GBA (Shi et al. 2024).

Macronutrient effects: High‐protein diets increase the abundance of protein‐fermenting bacteria (e.g., *Fusobacterium*, *Clostridium*), whereas high‐fibre diets favour saccharolytic bacteria (e.g., *Prevotella*, *Bacteroides*) that produce neuroprotective SCFAs. Raw meat‐based diets have been associated with increased microbial diversity but also carry potential pathogen risks (Zha et al. 2024).

Functional ingredients: Medium‐chain triglyceride (MCT) supplementation has shown cognitive benefits in senior dogs, likely through ketone body production. Tryptophan‐enriched diets may support serotonin synthesis, with preliminary evidence suggesting anxiolytic effects.

Practical implications: Unlike pharmaceuticals, dietary interventions are easily integrated into daily care, making them attractive adjunctive strategies for managing behavioural disorders in companion animals.

## 4 Clinical Applications and Evidence in Companion Animals

### 4.1 Evidence Grading Note

The evidence presented in this section is graded according to a framework adapted from the GRADE approach (Fletcher et al. 2024; Sargeant et al. 2022). The highest quality evidence comes from randomized controlled trials in client‐owned dogs. Where such trials are lacking, we have specified the evidence as ‘preliminary’ or ‘low’. Readers should interpret ‘moderate’ evidence as derived from small clinical trials requiring confirmation, and ‘very low’ evidence as hypothesis‐generating only (Fletcher et al. 2024; Sargeant et al. 2022).

The translation of GBA research into clinical veterinary practice is yielding promising, evidence‐based interventions for managing neurological and behavioural disorders in companion animals. A growing body of clinical evidence supports the use of microbiome‐targeted strategies as both standalone and adjunctive therapies.

### 4.2 Psychobiotics for Behavioural Disorders

Psychobiotics—live probiotics and prebiotics with mental health benefits—represent a leading clinical application. Specific strains have demonstrated significant efficacy in canine patients. For instance, *Bifidobacterium longum* BL999 and *Lactiplantibacillus plantarum* PS128 have been shown to reduce anxiety‐related behaviours and physiological stress markers, such as cortisol, in dogs (Sacoor et al. 2024). The therapeutic action of these psychobiotics involves multiple mechanisms: enhancing gut barrier integrity via SCFA production, regulating key neurotransmitter pathways (e.g., serotonin and GABA) and attenuating hyperactivity of the HPA axis and pro‐inflammatory cytokine release (Sacoor et al. 2024; Gorzelanna and Miszczak 2024). Clinical outcomes include measurable improvements in separation anxiety, aggression and fear responses, offering a well‐tolerated, non‐pharmacological option to complement conventional behavioural therapies (Sacoor et al. 2024).

### 4.3 Managing Canine Cognitive Dysfunction (CCD) and Epilepsy

GBA modulation holds significant promise for age‐related neurological conditions. CCD, a neurodegenerative condition analogous to Alzheimer's disease in humans, is a key target. Dietary interventions, prebiotics and probiotics that modulate the gut microbiota represent a logical therapeutic strategy to restore microbial balance, reduce inflammation and decrease the production of neurotoxic metabolites, potentially slowing the progression of CCD (Ambrosini et al. 2019; Wiley et al. 2017). Specifically, MCT supplementation has shown significant improvements in cognitive functions like memory and attention in senior dogs (Gorzelanna and Miszczak 2024).

For canine epilepsy, particularly refractory cases, therapeutic strategies aim to correct microbial dysbiosis to modulate neuroinflammation and seizure susceptibility. This includes using specific probiotics (e.g., *Lactobacillus* and *Bifidobacterium* strains) to restore inhibitory neurotransmission via GABA production and employing prebiotics to enhance SCFA production, which supports blood–brain barrier integrity (Gernone et al. 2022). More advanced interventions like FMT are being explored to correct dysbiosis‐induced immune activation, specifically targeting pro‐inflammatory pathways implicated in epileptogenesis (Gernone et al. 2022; Gorzelanna and Miszczak 2024).

### 4.4 The Role of FMT

FMT is an investigational approach with preliminary evidence for GBA‐targeted therapy in veterinary medicine. To date, most data derive from rodent models, but growing clinical application suggests that FMT can ameliorate neurological and behavioural disorders by restoring a healthy microbial balance and reducing systemic inflammation (Nishigaki et al. 2025). In dogs, FMT has been successfully piloted to alleviate behavioural comorbidities, such as ADHD‐like and fear‐related behaviours, in cases of drug‐resistant epilepsy, with outcomes correlating with beneficial shifts in neurotransmitters like GABA (Gorzelanna and Miszczak 2024). Although these preliminary results warrant further investigation, species‐specific studies are needed to establish standardized safety and efficacy protocols for FMT in veterinary practice (Nishigaki et al. 2025).

### 4.5 Translational Evidence and Future Directions

Although direct evidence from large‐scale clinical trials in pets is still accumulating, robust translational data from human and rodent studies strongly support the therapeutic potential of these approaches. Probiotics, such as *Bifidobacterium* (e.g., *B. longum*, *B. breve*) and *Lactobacillus* (e.g., *L. helveticus*, *L. rhamnosus*), have demonstrated efficacy in reducing anxiety‐ and depression‐like behaviours and improving cognitive function in preclinical models, primarily through modulation of the HPA axis, increased BDNF expression and altered neurotransmitter levels (Wang et al. 2016). The close resemblance of the canine gut microbiome to that of humans further validates the dog as a translationally relevant model and a direct beneficiary of this research (Ambrosini et al. 2019). The overarching aim of these clinical applications is to leverage microbiome science to offer novel, non‐pharmacological strategies for improving the mental health and overall welfare of companion animals (Wiley et al. 2017).

## 5 Challenges and Future Directions

The pursuit of GBA‐targeted therapies for companion animals, while promising, is confronted by several significant challenges that must be addressed to advance the field. A primary obstacle is the pervasive reliance on correlative data, which falls short of establishing definitive causal relationships between microbial dysbiosis and neurobehavioural pathologies (Kiełbik and Witkowska‐Piłaszewicz 2024; Gorzelanna and Miszczak 2024). This ‘chicken‐or‐egg’ dilemma—whether dysbiosis drives pathology or is a consequence of it—remains a fundamental knowledge gap (Homer et al. 2023). Furthermore, methodological limitations impede progress. Many studies depend on small sample sizes, partial 16S rRNA sequencing that lacks species‐level resolution and the generalization of complex gut ecosystems from single faecal samples, thereby hindering a precise understanding of microbial community dynamics and function (Homer et al. 2023; Shah et al. 2024; Blanquet et al. 2025). The clinical translation of findings is also hampered by an over‐reliance on behavioural assessments without the integration of comprehensive physiological biomarker panels, including cytokines, metabolites and neurotransmitters, which are essential for objective evaluation (Sacoor et al. 2024; Kiełbik and Witkowska‐Piłaszewicz 2024).

### 5.1 Key Research Gaps and Methodological Hurdles

Future studies must now uncover the exact ways the gut microbiome, through substances it produces and its effect on inflammation, communicates with the brain to shape behaviour (Kiełbik and Witkowska‐Piłaszewicz 2024; Ma et al. 2019). Another complicating factor is the realization that many conventional drugs used to treat CNS disorders may possess unintended antimicrobial properties, potentially disrupting the gut microbiome and creating a complex, counterproductive feedback loop, a consideration that must be incorporated into future drug discovery pipelines (Long‐Smith et al. 2020). Finally, the development of accessible, standardized and cost‐effective diagnostic tools for gut dysbiosis is urgently required to facilitate the transition of GBA diagnostics from research settings to routine veterinary practice (Naufel et al. 2023). A further limitation of the current literature is the lack of standardized dosing regimens, treatment durations and clinical protocols across studies, which hinders direct clinical translation and comparability between trials. The major research challenges and proposed solutions for advancing GBA research in companion animals are outlined in Table 3.

**TABLE 3:** Major research gaps and proposed solutions in canine and feline gut–brain axis research.

Research gap	Current limitation	Proposed solution	Expected outcome
Causality establishment	Correlative data predominates Chicken‐egg dilemma	Longitudinal studies Germ‐free animal models FMT intervention studies	Mechanistic understanding Targeted therapeutic development
Methodological standardization	16S rRNA sequencing limitations Sample variability	Multi‐omics approaches Standardized protocols Multiple sampling sites	Accurate microbial profiling Reproducible results
Species‐specific data	Limited feline studies Breed‐specific variations	Species‐focused trials Breed‐stratified analysis Feline‐specific probiotics	Tailored interventions Improved clinical outcomes
Clinical translation	Small sample sizes Limited biomarkers	Large‐scale clinical trials Biomarker panels development Multi‐centre collaborations	Evidence‐based protocols Personalized treatment approaches
Long‐term safety	Unknown long‐term effects Interaction with conventional drugs	Long‐term follow‐up studies Drug‐microbiome interaction screening	Safety profiles establishment Combination therapy optimization

### 5.2 A Framework for Future Research

There is a pressing need for rigorous, evidence‐based clinical trials, particularly double‐blind, placebo‐controlled studies, to validate the safety and efficacy of interventions like specific probiotics, prebiotics and FMT (Homer et al. 2023; Blanquet et al. 2025). To truly understand how the gut affects health, future research should use advanced tools that analyse not just which microbes are present, but what they are actually doing (Shah et al. 2024; Ma et al. 2019). To turn these findings into real‐world treatments, future studies should move beyond a one‐size‐fits‐all approach. By considering each animal's unique biology—like its native gut microbes, age and diet—we can create targeted strategies to improve behaviour through the GBA (Homer et al. 2023; Ma et al. 2019).

### 5.3 Publication Bias and Safety Considerations

A critical limitation of the current literature is the high risk of publication bias. Studies reporting positive effects of probiotics or FMT are more likely to be published than those with null or negative findings (Sargeant and O'Connor 2020). Unlike human medicine, veterinary research lacks registered clinical trial repositories that allow assessment of unpublished negative results, making it difficult to accurately estimate true effect sizes (Sargeant and O'Connor 2020).

Regarding safety, although psychobiotics are generally well‐tolerated with minimal side effects, FMT carries documented risks, including pathogen transmission, potential disruption of recipient microbial ecology and unknown long‐term consequences (Keay et al. 2026). Regulatory frameworks for FMT in veterinary practice remain underdeveloped, and standardized donor screening protocols are urgently needed before widespread clinical adoption (Keay et al. 2026). Clinicians should therefore view FMT as an investigational procedure rather than a standard clinical therapy.

## 6 Conclusion

The burgeoning field of GBA research heralds a transformative era in veterinary medicine, particularly for managing neurological and behavioural disorders in companion animals. Evidence from mechanistic studies and clinical applications consistently demonstrates that pharmacological modulation of the microbiome—through psychobiotics, specialized diets and microbiome restoration therapies—offers a viable and promising alternative or adjunct to conventional treatments. The efficacy of specific interventions in reducing anxiety, improving cognitive function and managing seizure activity underscores the therapeutic potential of targeting this bidirectional communication system.

However, the translation of these findings into widespread clinical practice faces substantial hurdles. The field must overcome methodological limitations in microbiome analysis, establish causal rather than correlative relationships between dysbiosis and pathology and develop standardized, accessible diagnostic protocols. Furthermore, the complex interplay between conventional pharmaceuticals and the microbiome necessitates a more nuanced approach to drug development and prescription.

Advancing the field will require (1) large‐scale, longitudinal studies to establish causality; (2) standardized, multi‐omics approaches for microbial profiling; (3) rigorous, placebo‐controlled clinical trials; and (4) species‐specific safety data, particularly for FMT. Until these gaps are addressed, microbiome‐targeted therapies should be viewed as adjunctive rather than replacement treatments. With continued rigorous investigation, the GBA holds genuine potential to expand the therapeutic options for veterinary behavioural medicine (Sargeant and O'Connor 2020).

## Author Contributions

**Sakineh Khanamani Falahatipour**: conceptualization, investigation, supervision, validation, writing – review and editing. **Motahareh Soltani**: resources, validation, visualization, methodology, writing – original draft.

## Funding

The authors have nothing to report.

## Disclosure

We confirm that all authors meet the journal's criteria for authorship. Each individual made substantial contributions to the work's conception, design, data interpretation, and manuscript preparation. All authors have reviewed and approved the final manuscript and accept accountability for the entire work.

## Conflicts of Interest

The authors declare no conflicts of interest.

## Ethics Statement

The authors have nothing to report.

## Data Availability

Data sharing not applicable to this article as no datasets were generated or analysed during the current study.
